# Capping Protein Regulator and Myosin 1 Linker 3 (CARMIL3) as a Molecular Signature of Ischemic Neurons in the DWI-T2 Mismatch Areas After Stroke

**DOI:** 10.3389/fnmol.2021.754762

**Published:** 2021-12-16

**Authors:** Shin-Joe Yeh, Pang-Hung Hsu, Ti-Yen Yeh, Wei-Kang Yang, Ko-Ping Chang, Chien-Sung Chiang, Sung-Chun Tang, Li-Kai Tsai, Jiann-Shing Jeng, Sung-Tsang Hsieh

**Affiliations:** ^1^Graduate Institute of Anatomy and Cell Biology, National Taiwan University College of Medicine, Taipei, Taiwan; ^2^Department of Neurology, National Taiwan University Hospital, Taipei, Taiwan; ^3^Department of Bioscience and Biotechnology, National Taiwan Ocean University, Keelung, Taiwan; ^4^Department of Pathology, National Taiwan University Hospital, Taipei, Taiwan; ^5^Department of Neurology, National Taiwan University Hospital Hsin-Chu Branch, Hsinchu, Taiwan; ^6^Graduate Institute of Clinical Medicine, National Taiwan University College of Medicine, Taipei, Taiwan; ^7^Graduate Institute of Brain and Mind Sciences, National Taiwan University College of Medicine, Taipei, Taiwan

**Keywords:** ischemic stroke, DWI-T2 mismatch, DWI-FLAIR mismatch, capping protein regulator and myosin 1 linker 3, CARMIL3, edema, oxidative stress

## Abstract

Ischemic stroke with a mismatch between diffusion-weighted imaging (DWI) and fluid-attenuated inversion recovery (FLAIR) or T2-weighted images indicates onset within 4.5 h, but the pathological substrates in the DWI-T2 mismatch and T2(+) areas remain elusive. In this study, proteomics was used to explore (1) the protein expression profiles in the T2(+), mismatch, and contralateral areas, and (2) the protein with the highest expression in the T2(+) area in the brains of male Sprague-Dawley rats within 4.5 h after middle cerebral artery occlusion (MCAO). The expression of the candidate protein was further validated in (1) rat brain subjected to MCAO, (2) rat primary cortical neuronal culture with oxygen-glucose deprivation (OGD), and (3) infarcted human brain tissues. This study showed that apoptosis was observed in the T2(+) and mismatch regions and necroptosis in the T2(+) region of rat brains after MCAO. We identified capping protein regulator and myosin 1 linker 3 (CARMIL3) as the candidate molecule in the T2(+) and mismatch areas, exclusively in neurons, predominantly in the cytoplasm, and most abundant in the mismatch area. The CARMIL3(+) neurons and neurites in the mismatch and T2(+) areas were larger than those in the control area, and associated with (1) increased expression of sulfonylurea receptor 1 (SUR1), indicating edema, (2) accumulation of p62, indicating impaired autophagy, and (3) increase in 8-hydroxy-2′-deoxyguanosine (8-OHdG), indicating oxidative stress. The increased expression of CARMIL3 was validated in a cell model of cortical neurons after OGD and in infarcted human brain tissues. In conclusion, this study shows that the mismatch and T2(+) areas within 4.5 h after ischemia are characterized by upregulated expression of CARMIL3 in neurons, particularly the mismatch area, which is associated with neuronal edema, impaired autophagy, and oxidative stress, indicating that CARMIL3 serves as a molecular signature of brain ischemia.

## Introduction

In ischemic stroke, the phenomenon with positive signals on diffusion-weighted imaging (DWI) but negative signals on fluid-attenuated inversion recovery (FLAIR) is defined as DWI-FLAIR mismatch, which indicates ischemia within 4.5 h (Thomalla et al., 2011). Clinically, this pattern serves as a selection criterion for thrombolysis in ischemic stroke patients with unclear onset time (Thomalla et al., 2018). Given that DWI vs. FLAIR or T2-weighted images (T2) reflect cytotoxic vs. vasogenic edema, a critical issue is the molecular substrate of ischemic neurons underlying the mismatch between these image patterns during the earliest stage of ischemia.

Neurons are extremely sensitive to ischemia, and the expression patterns of several molecules are altered in ischemic neurons. Structural proteins such as microtubule-associated protein 2 (MAP2) and neuronal nuclear antigen (NeuN) have reduced immunoreactivity within 12 and 24 h, respectively, after middle cerebral artery occlusion (MCAO) (Buscemi et al., 2019). Regarding ischemia-induced cellular edema, sulfonylurea receptor 1 (SUR1) regulates this edema by controlling a cation channel that is opened by ischemia, with higher expression in the infarct core than in the peri-infarct region after MCAO (Simard et al., 2006). Free radicals contribute to the ischemia-induced cellular edema; 8-hydroxy-2′-deoxyguanosine (8-OHdG) is a hallmark of oxidative DNA damage that is increased in the peri-infarct regions but not in the infarct core at 24 h following pMCAO (Nagayama et al., 2000). However, these molecules with increased expression after cerebral ischemia are not specific for neurons, and rare studies focused on the changes within the first few hours.

In the early hours of ischemia, the areas of DWI-T2 mismatch and DWI-T2 positive [T2(+) area] can coexist, with the degree of edema in the T2(+) area being more severe than that in the mismatch area (Neumann-Haefelin et al., 2000). Previous proteomic studies were based on anatomical or morphological definitions of ischemic areas, that is, sampling the cortex of the ischemic hemisphere as the ischemic area or regarding the striatum as the infarct core (Chen et al., 2007; Zgavc et al., 2013). Few studies have applied MRI such as DWI to define ischemic brain areas in animal models of stroke (Wu et al., 2017). There has also been a lack of systematic phenotyping of the mismatch area at the molecular level. To achieve this goal, we applied high-throughput proteomics to explore the molecular signatures in the mismatch vs. T2(+) areas in a MCAO model of rats (with MRI acquisition within 4.5 h after ischemia), comparing them to those of the corresponding areas on the contralateral side (control areas). In addition to using permanent MCAO (pMCAO) to imitate an irreversible acute occlusion in cerebral arteries, we took advantage of transient MCAO (tMCAO) with rapid recanalization to mimic a rescue intervention. This finding was further validated in infarcted human brain tissues and primary cortical neuronal cultures exposed to oxygen-glucose deprivation (OGD).

## Materials and Methods

### Animals

Male Sprague-Dawley rats, weighing between 250–275 g, were purchased from BioLASCO Taiwan Co., Taiwan, and housed with free access to food and water under diurnal lighting conditions (12 h of darkness and 12 h of light). The animal experiments were approved by the Institutional Animal Care and Use Committee of the National Taiwan University College of Medicine.

### Middle Cerebral Artery Occlusion Model

Permanent and transient MCAO (pMCAO or tMCAO) procedures were performed on rats to mimic human ischemic stroke without and with recanalization, respectively. The MCAO model was induced by occlusion of the right middle cerebral artery using a silicon-coated nylon filament (MCAO sutures, RWD Life Science, Shenzhen, China), as previously described (Yeh et al., 2018). The filament was not removed in the pMCAO group and was removed after 90 and 30 min in the tMCAO-90 and tMCAO-30 groups, respectively. In addition, a sham group received similar surgical procedures as MCAO groups but without occlusion of MCA.

### MRI-Based Brain Tissue Collection

Brain MRI was performed at 3 h after pMCAO, tMCAO, or sham operation (*n* = 29; 4 for proteomics experiment and 25 for immunohistochemical study) using 7T MRI machine (Bruker PharmaScan, Ettlingen, Germany) at National Taiwan University to acquire the T2-weighted images (T2) and DWI (Yeh et al., 2018). After MRI acquisition, the brain for proteomics experiment was harvested following intracardiac perfusion with 1% sodium nitrite under anesthesia. Based on the MR images, we collected three regions of brain tissues: T2(+) (positive signals on both DWI and T2), mismatch (positive signals on DWI but not on T2), and the corresponding regions in the contralateral hemisphere. The positive regions on T2 or DWI were determined manually on ImageJ software (National Institute of Mental Health, Bethesda, MD, United States) according to the higher gray values with the difference larger than 2000 compared with those of the contralateral corresponding regions. Lesion areas were outlined and measured on each slice, and lesion volumes were calculated by summation of these lesion areas multiplied by the thickness. The control group included four naïve rats in the proteomics analysis and the brain tissues within the middle cerebral artery territory were collected.

### Proteomics Analysis

We used proteomics to analyze the brain tissues collected from different regions according to MR images. These collected brain tissues were homogenized in lysis buffer (8 M urea in 50 mM triethyl ammonium bicarbonate (TEAB) buffer, pH 8), then centrifuged at 16,000 × *g* for 10 min at 4°C. The supernatant was collected as the protein sample, and protein concentrations were determined by BCA protein assay (Thermo Fisher Scientific, United States). Each protein sample (100 μg) was transferred for Tandem Mass Tag (TMT) isobaric labeling (Thermo Fisher Scientific) to perform quantitative proteomics analysis according to the manufacturer’s instructions. In brief, protein samples were reduced by 200 mM tris (2-carboxyethyl) phosphine (TCEP) at 55°C for 1 h, followed by the addition of 375 mM iodoacetamide to perform the alkylation reaction at room temperature in the dark for 30 min. Six volumes of pre-chilled (−20°C) acetone were added to each sample, followed by freezing at −20°C for at least 4 h to precipitate the proteins. The acetone-precipitated protein pellet was collected by centrifugation and then resuspended in 50 mM triethylammonium bicarbonate (TEAB) and trypsin (protein/trypsin ratio of 50:1) for proteolysis overnight at 37°C. The digested samples were transferred to the corresponding TMT reagent vial for labeling at room temperature for 1 h. The TMT labeling reaction was terminated by the addition of 8 μL of 5% hydroxyamine. Equal amounts of TMT-labeled peptide samples from the corresponding regions of the four animals were combined, and a high pH reversed-phase peptide fractionation kit (Thermo Fisher Scientific) was used to fractionate samples before LC-MS/MS analysis.

LC-MS/MS analysis of the fractionated peptide samples was performed on an Orbitrap Fusion mass spectrometer (Thermo Fisher Scientific Inc.) equipped with an EASY-Spray LC column (50 μm I.D. ×150 mm, particle size 2 μm, pore size 100 Å). The chromatographic separation of peptides was performed using 0.1% formic acid in water (mobile phase A) and 0.1% formic acid in 80% acetonitrile (mobile phase B) at a flow rate of 300 nL/min for 120 min. The electrospray voltage was maintained at 1.8 kV in the positive ion mode, and the capillary temperature was set at 275°C. The MS survey scans were operated in a mass range of m/z 350–1,600 (automatic gain control, AGC target 5e5), with a mass accuracy of <5ppm, a resolution of 120,000, and a maximum injection time of 50 ms. The target m/z was isolated and performed for data-dependent MS/MS scan by assisted HCD with collision energies of 31, 35, and 39%, 15,000 resolution, and 200 ms maximum injection time. The AGC target 5e4 was set to trigger MS/MS analysis with the previously selected ion dynamically excluded for 60 s.

Quantitative proteomics analysis of the MS RAW data was performed using MaxQuant (ver. 1.6.7.0) with the rat protein database obtained from UniProt reviewed *Rattus norvegicus* proteomes. For database searches, the precursor mass tolerance was set to 20 ppm for the first search and 4.5 ppm for the main database search. The fragment ion mass tolerance was set to 0.5 Da. Trypsin was chosen as the enzyme, and two missed cleavages were allowed. Carbamidomethylation of cysteine was defined as a fixed modification, and methionine oxidation was defined as variable modifications. The minimum peptide length was set to seven amino acids, and the minimum number of unique peptides was set to one. The maximum false discovery rate (FDR), calculated by employing a reverse database, was set to 5% for both peptides and proteins. Proteins identified as “reverse” and “only identified by site” were discarded from the list of identified proteins. The corrected intensities of the TMT tag signals were used for protein quantitation.

To analyze the proteomics data, hypoxanthine-guanine phosphoribosyltransferase (HPRT) was used as an internal control to normalize the protein levels due to its stable expression in ischemic lesions (Kang et al., 2018). Then, the expression levels of individual proteins in normal brain tissues were used as reference to calculate the ratios of expression levels in different regions (infarct, mismatch, and contralateral regions).

The functions and subcellular locations of all the identified proteins were analyzed to show the profiling. Capping protein regulator and myosin 1 linker 3 (CARMIL3) was the protein with the highest expression in the T2(+) region and increased expression in the mismatch area; thus, this finding was validated by immunofluorescence experiments.

### Immunofluorescence Staining of Rat Brain Tissues

The rats that underwent pMCAO, tMCAO, or sham procedures followed by brain MRI studies received transcardial perfusion fixation with 4% paraformaldehyde. After post-fixation and immersion in 30% sucrose solution, the brain tissues were sliced into 14 μm-sections using a cryostat microtome. The sections were washed with 0.5 M Tris buffer and blocked with 0.5% Triton-milk buffer (0.5% non-fat milk power and 0.5% Triton X-100 in 0.5 M Tris buffer), then immunofluorescence staining was conducted to evaluate the expression pattern of several molecules including CARMIL3 (1:50, No. 037943, United States Biological), neuronal markers such as NeuN (1:100, No. M-377-100, Biosensis) and MAP2 (1:500, No. PA1-16751, Invitrogen), glial marker GFAP (1:200, No. ab53554, Abcam), microglial marker Iba1 (1:50, No. ab107159, Abcam), edema marker SUR1 (1:100, No. NBP2-59320, Novus), autophagy marker p62 (1:300, No. ab56416, Abcam), oxidative stress marker 8-OHdG (1:200, No. ab5830, Abcam), necroptosis marker phosphorous receptor-interacting protein kinase 3 (pRIP3) (1:100, No. PA5-105701, Thermo Fisher Scientific), and apoptosis marker active caspase 3 (1:50, No. A36943, antibodies.com), diluted in 0.5% triton-milk buffer and incubated at 4°C overnight. After washing, the specimens were incubated for 1 h with secondary antibodies (all 1:500) diluted in 0.5% Triton-milk buffer. DAPI was used to stain the nuclei. A confocal microscope (Leica TCS SP8 × STED 3×) was used to capture the images of the T2(+), mismatch, and contralateral areas.

Another group of rats (*n* = 4) received brain MRI study at 24 h after pMCAO and sacrificed on day 3 after stroke. The brain tissues were processed in the same way as aforementioned and CARMIL3 immunofluorescence staining was performed to clarify the changes of CARMIL3 in T2(+) regions at a later stage corresponding to the timing of obtaining human infarct brain tissues by decompressive surgery.

### Immunohistochemical Staining of Human Brain Tissues

The infarcted human brain tissues were obtained from decompressive surgery for large cerebral infarcts, and the normal brain tissues were obtained from autopsies of stroke-free subjects; both tissues were fixed using formalin and embedded in paraffin. Experiments had been approved by the Research Ethics Committee of the National Taiwan University Hospital. The sections were processed by deparaffinization with xylene, rehydration, and epitope retrieval with Leica Epitope Retrieval Solution pH6 (Leica Biosystems), followed by blocking hydrogen peroxide (No. TA-125-HP, Thermo Fisher Scientific). Protein blocking solution (Ultra V block, No. TA-125-UB, Thermo Fisher Scientific) was then applied on the sections. Next, the sections were incubated with CARMIL3 antibody (1:500, No. 037943, United States Biological) diluted in Ventana Antibody Dilution Buffer (No. ADB250, Ventana Medical Systems) for 1.5 h at room temperature, and then incubated with Primary Antibody Amplifier (No. TL-125-QPB, Thermo Fisher Scientific). After washing, horseradish peroxidase (HRP)-conjugated secondary antibody (No. TL-125-QPH, Thermo Fisher Scientific) was applied on the sections with incubation for 10 min. The sections were then incubated with 3,3′-diaminobenzidine (DAB) chromogen and substrate mixture solution (No. TA-004-QHCX and TA-125-QHSX, Thermo Fisher Scientific) for 3 min. Hematoxylin diluted in distilled water was used for counterstaining. Microscopic images were obtained using a light microscope (Olympus BX53, Japan).

### Primary Cortical Neuronal Culture and Oxygen-Glucose Deprivation

For the preparation of cortical neurons, pregnant Sprague-Dawley rats were killed by CO_2_ inhalation on embryonic day 16. The embryos were dissected to obtain brain cortices which were trypsinized, triturated and passed through a 40-μm cell strainer (FALCON, Corning, United States) to harvest neurons. The cells were seeded on poly-L-lysine (Sigma-Aldrich, St. Louis, MO, United States)-coated 100-mm-diameter cell culture dishes or coverslips-based dishes containing neuronal culture medium (Neurobasal medium, 2% B-27, 0.5 mM Glutamax, 1% penicillin–streptomycin, all from Life Technologies Corporation, United States). After 8-day cell culture, these cells were used for experiments. They were treated with oxygen-glucose deprivation (OGD) for 3 h followed by normoxia for 0, 3, or 24 h (Young et al., 2020). For the standard (normoxia) condition, cells were maintained under an ambient atmosphere in an incubator at 37°C with 5% CO_2_. For the induction of OGD, primary neurons were incubated in glucose-free Locke medium containing 154 mM NaCl, 5.6 mM KCl, 2.3 mM CaCl_2_ ⋅ 2H_2_O, 1.0 mM MgCl_2_ ⋅ 6H_2_O, 3.6 mM NaHCO_3_, and 5 mM 4-(2-hydroxyethyl)-1-piperazineethanesulfonic acid (HEPES) buffer (pH 7.2) in a hypoxic chamber containing 0.5% O_2_, 95% N_2_, and 5% CO_2_ for 3 h (Ruskinn InvivO_2_ 400 Workstation, United Kingdom). After the 3-h hypoxic challenge, the cultures were removed from the hypoxic chamber, and the OGD solution in the culture dishes was replaced with neuronal culture medium. The cells were then allowed to recover for 3 or 24 h in a humidified incubator at 37°C with 5% CO_2_ as normoxia condition, mimicking reperfusion. Next, the cell specimens were analyzed by western blot and immunofluorescence experiments.

### Immunofluorescence Staining of Cultured Cortical Neurons

The coverslip-based cell culture specimens were washed with 1× phosphate buffered saline (PBS), followed by incubation with 0.5% Triton-PBS solution for 30 min, and then incubation with primary antibodies including CARMIL3 (1:10000, No. 037943, United States Biological), p62 (1:1000, No. ab56416, Abcam), and MAP2 (1:1000, No. PA1-16751, Invitrogen), diluted in the buffer made by 1:1 10% bovine serum albumin (BSA) and 0.5% Triton-PBS, and incubated at 4°C overnight. After washing, the specimens were incubated with secondary antibody solution (all 1:500) mixed with phalloidin 633 dyes (1:1000, No. ab176758, Abcam). A confocal microscope (Leica TCS SP8 × STED 3×, United States) was used to capture the images.

### Western Blot

The expression levels of CARMIL3 of the specimens in the primary cortical neuron culture experiment were quantified by Western blot. While placed on ice and washed with ice-cold PBS, the cell culture plates were added with ice-cold lysis buffer (0.5 mL/100-mm dish, composed of 10× phosphatase inhibitor, 50× protease inhibitor, and 1× radioimmunoprecipitation assay [RIPA] buffer) and the attached cells were scraped off and transferred to an Eppendorf with maintaining frequent agitation for 30 min. After centrifugation, the supernatant was collected as protein solution. The protein concentrations were quantified by bicinchoninic acid (BCA) assay. The protein solution was denatured by heating at 100°C for 10 min after adding Laemmli sample buffer. Protein samples (10 μg per sample) were loaded onto a 8% SDS-PAGE gel, followed by electrophoresis. The separated proteins in the gel were transferred onto a PVDF membrane. The transferred membrane was blocked in 5% non-fat milk for 1 h, and then incubated with primary antibodies against CARMIL3 (1:1000, No. 037943, United States Biological), p62 (1:5000, No. ab56416, Abcam) and α-tubulin (1:5000, No. T9026, Sigma-Aldrich) at 4°C overnight, followed by incubation with secondary antibody for 1 h at room temperature. After incubating the membrane with an ECL chemiluminescence detection kit, the protein bands were detected using an X-ray film.

### Statistical Analysis

Microscopic images were analyzed using ImageJ software (National Institute of Mental Health, Bethesda, MD, United States); the region of interest (ROI) method was applied to define the boundaries of all the neurons in a picture. Positivity of a molecule [e.g., CARMIL3(+)] in a neuron was defined as the intensity higher than a threshold, which was “mean + 2 × standard deviation” of the intensities of all neurons in the contralateral areas of four rats. CARMIL3(+) neurites were similarly defined as the CARMIL3 intensity of a neurite higher than “mean + 2 × standard deviation” of CARMIL3 intensities of all identifiable neurites in the contralateral areas of four rats. For analyzing staining results, the density (number/image area) of positively stained neurons for a molecule was defined as positively stained neuron density, while the ratio of positively stained neurons in all neurons were defined as positively stained neuron ratio. Neuron ratios or neurite ratios were calculated because ischemia affected the staining intensity of NeuN and MAP2 which were used to define neurons and neurites, respectively, and the number of certain molecule (+) neurons or neurites might be underestimated due to loss of neuronal markers in some neurons. Student’s *t*-test and one- or two-way ANOVA tests were used to compare continuous variables. Linear regression was conducted to analyze the correlation between the intensities of CARMIL3 and p62 or 8-OHdG.

## Results

### MRI Findings in Rat Brains After Permanent Middle Cerebral Artery Occlusion or Transient Middle Cerebral Artery Occlusion

**FIGURE 1:**
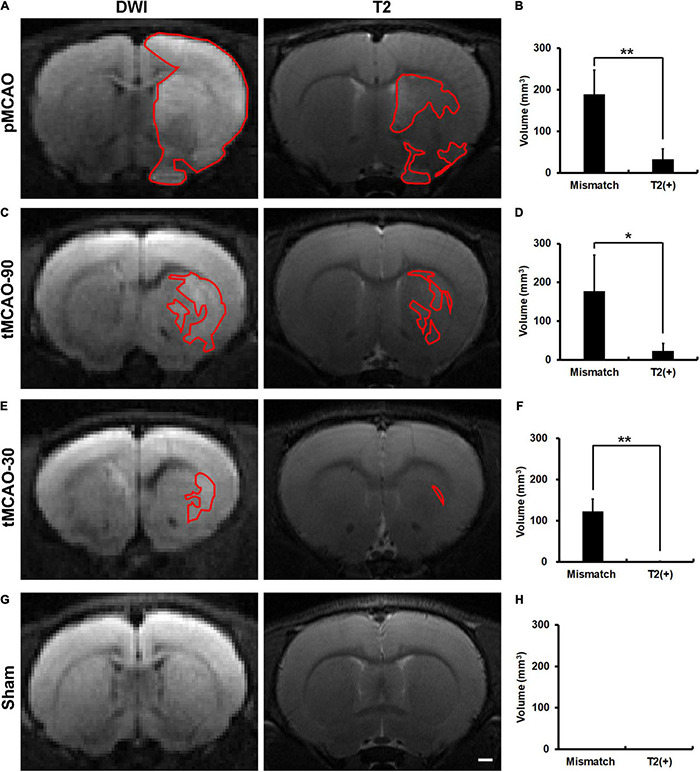
Brain MR images of rats, within 4.5 h after permanent middle cerebral artery occlusion (pMCAO), transient middle cerebral artery occlusion (tMCAO), or sham operation. **(A,C,E,G)** Representative images of DWI and T2 at the corresponding sections of rat brains after pMCAO **(A)**, tMCAO 90 min (tMCAO-90, **C**), tMCAO 30 min (tMCAO-30, **E**), and sham operation **(G)**. The areas outlined by red lines indicates the regions with hyperintensity. The hyperintense areas on both T2 and DWI were defined as T2(+) regions, while those with hyperintensity on DWI but not on T2 were the mismatch areas. **(B,D,F,H)** These graphs show the comparison of the volumes of mismatch and T2(+) regions among the pMCAO (**B**, *n* = 8), tMCAO-90 (**D**, *n* = 5), tMCAO-30 (**F**, *n* = 4), and sham (**H**, *n* = 4) groups. The volumes of the mismatch areas were significantly larger than those of the T2(+) areas in the three ischemic conditions (*p < 0.05; **p < 0.01). The T2(+) volumes in the tMCAO-30 group were significantly smaller than those in the pMCAO (*p* = 0.007) and tMCAO-90 groups (*p* = 0.014), while the T2(+) volumes were similar between the tMCAO-90 and pMCAO groups. **(G,H)** Sham-operated animals (*n* = 4) did not have hyperintensity lesions on DWI and T2 weighted images. Scale bar = 10 μm.

We first established a DWI-T2 mismatch platform and examined the neuroimaging patterns after pMCAO. The latency from MCAO to MRI acquisition was 166 ± 36 min, and that from finishing MRI acquisition to sacrifice was 46 ± 7 min. The mortality rate before sacrifice was 6% (1/17) in the pMCAO group. In contrast to the sham group, all rats in the pMCAO group had coexisting mismatch and T2(+) regions (Figure 1).

To investigate whether recanalization would alleviate the changes in neuroimaging patterns, we performed transient MCAO (tMCAO) for 90 min and 30 min as an intervention with different degrees of rescue. The mortality rate was 0% in the tMCAO group. All rats in the tMCAO-90 group and 75% of rats in the tMCAO-30 group had coexisting mismatch and T2(+) regions (Figure 1).

The mismatch volumes were significantly larger than the T2(+) volumes in the pMCAO and tMCAO groups (*p* < 0.05). The T2(+) volumes in the tMCAO-30 group were significantly smaller than those in the tMCAO-90 (0.6 mm^3^ vs. 23.3 mm^3^, *p* = 0.014) and pMCAO groups (0.6 mm^3^ vs. 32.8 mm^3^, *p* = 0.007), while the tMCAO-90 and pMCAO groups had similar T2(+) volumes. Furthermore, the volumes of the mismatch areas did not differ among the three groups.

### Cell Densities, Apoptosis, and Necroptosis in the T2(+) and Mismatch Regions

**FIGURE 2:**
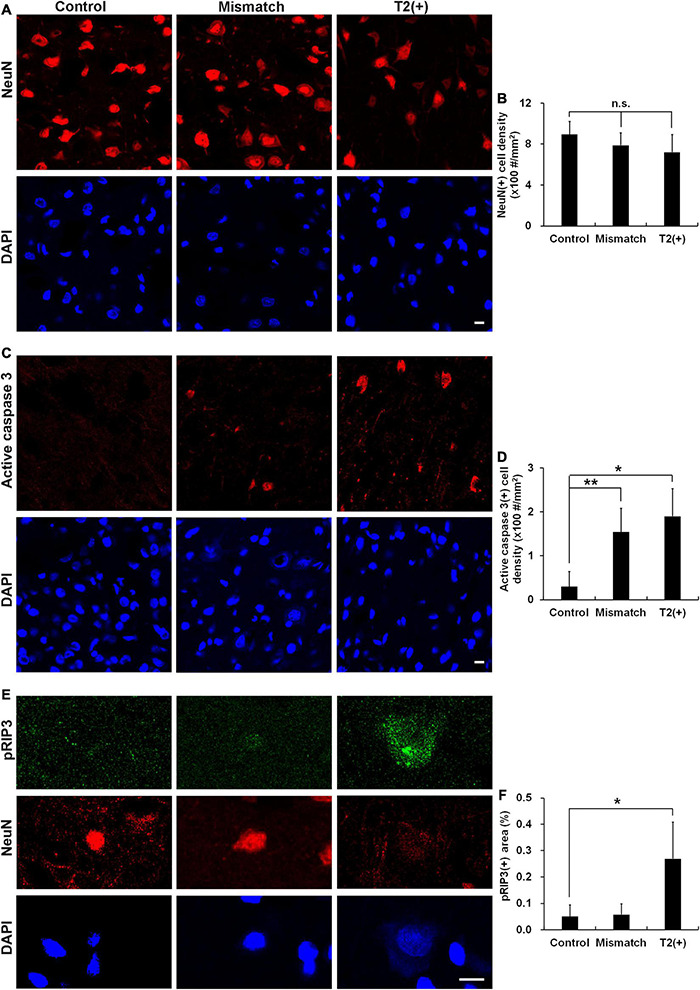
Cell densities, apoptosis, and necroptosis in the T2(+), mismatch, and control regions. **(A,B)** Staining results of NeuN and DAPI in the T2(+), mismatch, and control areas. NeuN(+) cell densities (#/mm^2^) were not different among the three regions (*p* = 0.399). **(C,D)** Staining results of active caspase 3 in the three regions. Active caspase 3 was observed in some neurons in the mismatch and T2(+) regions. The cell densities of active caspase 3(+) in the mismatch and T2(+) regions were significantly higher than those in the control regions (*p* = 0.009 and 0.019 by paired *t*-test, respectively; *p* = 0.008 for comparison of the three regions). **(E)** The expression of pRIP3 was obvious in some swollen neurons in the T2(+) regions. **(F)** The expression area proportion of pRIP3 [the area of pRIP3(+) divided by the total analyzed area] were significantly larger in the T2(+)regions than in the control areas (*p* = 0.022 by paired *t*-test; *p* = 0.021 for comparison of the three regions). *p < 0.05; **p < 0.01. *N* = 4 rats in each group. Scale bars = 10 μm.

To understand the pathological patterns of the mismatch area, we first analyzed the expression of cell death by examining cell and neuron densities in the T2(+) and mismatch regions after a short duration of ischemia in the pMCAO group. Positively stained cell density was calculated as numbers of positively stained cells divided by total image area. The neuronal densities did not change in the T2(+) and mismatch regions compared with those in the control region, but the NeuN intensity in some neurons decreased in the T2(+) region (Figures 2A,B). We then investigated whether apoptosis and necroptosis were involved in these regions by examining the expression of active caspase 3 and phosphorylated receptor-interacting protein 3 (pRIP3), respectively. The densities of active caspase 3(+) cells indicating apoptosis in the mismatch and T2(+) areas were significantly higher than those in the control area (Figures 2C,D). The pRIP3(+) area proportion [the area of pRIP3(+) divided by the total analyzed area] was larger in the T2(+) region than in the control region (Figures 2E,F).

### Categorization of the Proteins Retrieved From the Proteomics Experiment

To explore the protein expression patterns in ischemic regions, we applied proteomics experiments. The proteomics analysis identified 3008 proteins, and the data are available via ProteomeXchange with identifier PXD027890. The major functions and subcellular localizations of all the proteins were summarized in Supplementary Table 1. The three major functional categories were catalytic activity (*n* = 994), binding activity (*n* = 750), and signaling pathway regulation (*n* = 210). These proteins were most frequently distributed in the cytosol (*n* = 1572), nucleus (*n* = 1226), and plasma membrane (*n* = 1102). The top 10 abundance proteins in T2(+) region were shown in Table 1. The protein with the highest expression ratio in the T2(+) region was CARMIL3 (T2(+)/naïve ratio = 29.24, mismatch/naïve ratio = 2.88). We then conducted the following experiments to verify the expression and characterize the cell biology of CARMIL3 in ischemic stroke.

**TABLE 1:** Top 10 abundance proteins in T2(+) regions.

Rank	Protein ID	Protein name
1	Q5XHY1	Capping protein regulator and myosin 1 linker 3
2	Q9JHU5	Arfaptin-1
3	P81799	N-acetyl-D-glucosamine kinase
4	Q5I0E6	RNA polymerase II subunit B1 CTD phosphatase Rpap2
5	Q5XI37	28S ribosomal protein S15, mitochondrial
6	Q63796	Mitogen-activated protein kinase kinase kinase 12
7	Q56AP7	Protein cereblon
8	D4ACX8	Protocadherin-16
9	Q5PT56	Sodium/bile acid cotransporter 4
10	P02767	Transthyretin

### Capping Protein Regulator and Myosin 1 Linker 3(+) Neurons With Larger Cell Bodies and Neurites

**FIGURE 3:**
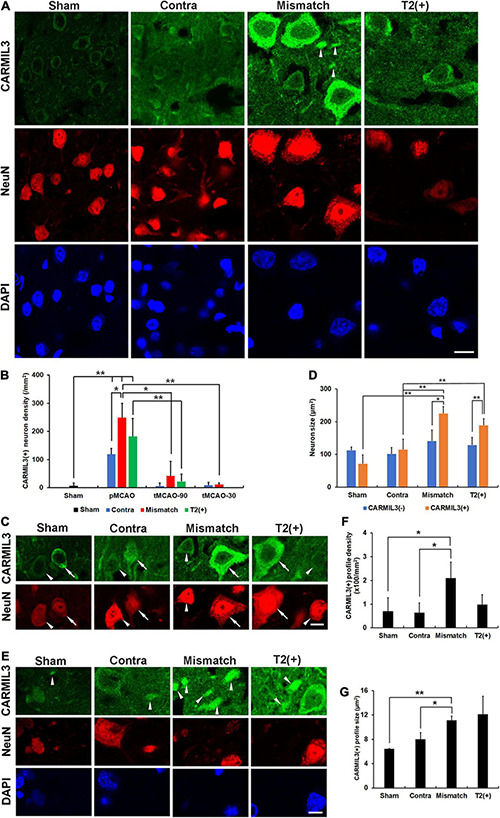
CARMIL3 expression pattern. **(A)** Expression of CARMIL3 and NeuN in the T2(+), mismatch, contralateral regions of pMCAO groups and sham group. In addition to being expressed in neuronal cytoplasm, CARMIL3 was also expressed in some cell parts without direct connection with neuronal bodies, named CARMIL3(+) profiles (arrowhead). **(B)** CARMIL3(+) neuron densities at different ischemic cortical regions in pMCAO and tMCAO of rat brains. The expression of CARMIL3 in the T2(+) region after tMCAO-30 was not quantified due to nearly lack of cortical T2(+) area. There was a significant difference in CARMIL3(+) neuron densities among the three regions after pMCAO (*p* = 0.028), and all of them were significantly higher than that in the sham group (all *p* < 0.01). CARMIL3(+) neuron densities in the mismatch regions after pMCAO were higher than those in the contralateral areas (*p* = 0.026) and mismatch regions after tMCAO-90 (*p* = 0.044) or tMCAO-30 (*p* = 0.002). The CARMIL3(+) neuron densities at mismatch regions were significantly different across the three different ischemic durations (*p* < 0.0001). The CARMIL3(+) neuron densities at the T2(+) regions after tMCAO-90 were significantly reduced than those after pMCAO (*p* = 0.003). **(C,D)** Representative images of CARMIL3(+) (arrows) and CARMIL3(−) neurons (arrowheads) at the three regions in pMCAO brains, showing that CARMIL3(+) neurons were bigger than CARMIL3(−) neurons in the T2(+) and mismatch regions. Moreover, CARMIL3(+) neurons at the T2(+) or mismatch regions were significantly bigger than those in contralateral areas. Two-way ANOVA analysis showed regions (*p* = 0.017) and CARMIL3 positivity (*p* = 0.018) significantly affected neuron sizes. **(E)** Representative images of CARMIL3(+) profiles which were more prominent in the mismatch regions. **(F,G)** The CARMIL3(+) profiles in the mismatch regions were more abundant **(F)** and larger **(G)** than those in the contralateral regions or sham group (all *p* < 0.05). Contra, contralateral. Scale bars = 10 μm. *N* = 4 rats in each group. *p < 0.05; **p < 0.01.

**FIGURE 4:**
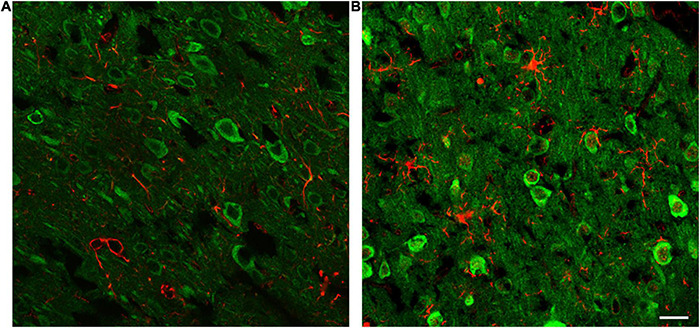
CARMIL3 expression in glial and microglial cells. Double-labeling staining of CARMIL3 with GFAP **(A)** and Iba1 **(B)** in the mismatch regions showed that CARMIL3 was not colocalized with glial or microglial cells. Scale bar = 25 μm.

To investigate the pathological significance of CARMIL3, we analyzed its expression patterns in the rat stroke brain. CARMIL3 was highly expressed in the neuronal cytoplasm in the T2(+) and mismatch regions compared to the contralateral region and sham group (Figure 3A). There was a significant difference of CARMIL3(+) neuron densities among the three regions after pMCAO (*p* = 0.028), and all of them were significantly higher than that in the sham group (all *p* < 0.01) (Figure 3B). The densities of CARMIL3(+) neurons in the mismatch areas were significantly higher than those in the contralateral areas in the pMCAO group (*p* < 0.05). Furthermore, glial or microglial cells did not have CARMIL3(+) features (Figure 4).

We then applied tMCAO models (tMCAO-90 and tMCAO-30 groups) to explore the effect of recanalization on CARMIL3 expression. The CARMIL3(+) neuron densities in mismatch regions were significantly different among the three different ischemic durations (*p* < 0.0001) (Figure 3B). There were fewer CARMIL3(+) neurons in the mismatch areas of the tMCAO-90 and tMCAO-30 groups than in those of the pMCAO group (*p* < 0.05 and *p* < 0.01, respectively). The CARMIL3(+) neuron densities in the T2(+) areas after tMCAO-90 were significantly lower than those after pMCAO (*p* < 0.01). Overall, two-way ANOVA analysis showed that CARMIL3(+) neuronal densities were significantly affected by ischemic durations (*p* = 0.0002) and regions (*p* < 0.0001).

The neuron sizes were significantly different by regions (*p* = 0.017) and CARMIL3 positivity (*p* = 0.018) after pMCAO, respectively (Figures 3C,D). Moreover, CARMIL3(+) neurons in the T2(+) and mismatch areas had larger cell bodies than CARMIL3(−) neurons in the same areas and CARMIL3(+) neurons in the contralateral areas, respectively (all *p* < 0.05). Interestingly, several CARMIL3(+) profiles without direct connection with neuronal bodies were colocalized with NeuN but not with DAPI (Figure 3E). The mismatch area had higher densities and larger sizes of CARMIL3(+) profiles than the contralateral areas and sham group (all *p* < 0.05) (Figures 3F,G). There was no difference in the densities or sizes of CARMIL3(+) profiles between contralateral regions and sham group.

**FIGURE 5:**
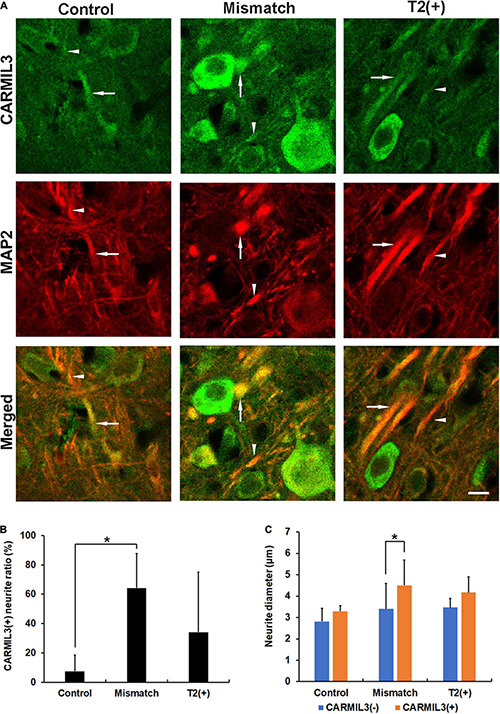
Expression pattern of MAP2 in the CARMIL3(+) profiles. **(A)** Double labeling of CARMIL3 and MAP2 in the T2(+), mismatch, and control regions after pMCAO. The CARMIL3(+) profiles were colocalized with MAP2, indicating that CARMIL3(+) profiles were located on neurites. CARMIL3(+) neurites (arrows) were thicker than CARMIL3(−) neurites (arrowheads) in the mismatch region. **(B)** CARMIL3(+) neurite ratio [the number of CARMIL3(+) neurites divided by that of all identifiable neurites] in the three regions. There were significantly higher ratios of CARMIL3(+) neurites in the mismatch regions compared with control areas (*p* = 0.035). **(C)** Comparison of the neurite diameters of CARMIL3(+) and CARMIL3(−) neurites. CARMIL3(+) neurites had significantly larger neurite diameters than CARMIL3(−) ones in mismatch regions (*p* = 0.036). *p < 0.05. *N* = 4 rats in each group. Scale bar = 10 μm.

Next, we performed double-labeling staining with CARMIL3 and MAP2 to clarify the nature of the CARMIL3(+) profiles. The colocalization of CARMIL3(+) profiles with MAP2(+) profiles confirmed the localization of these CARMIL3(+) profiles on neurites (Figure 5A). CARMIL3(+) neurite ratios [the number of CARMIL3(+) neurites divided by that of all identifiable neurites] were significantly higher in the mismatch areas than in the control area (*p* < 0.05) (Figure 5B). Furthermore, CARMIL3(+) neurites had larger diameters than CARMIL3(−) neurites in the mismatch area (*p* < 0.05) (Figure 5C).

### Relationship of Neuronal Capping Protein Regulator and Myosin 1 Linker 3 With Cell Edema

**FIGURE 6:**
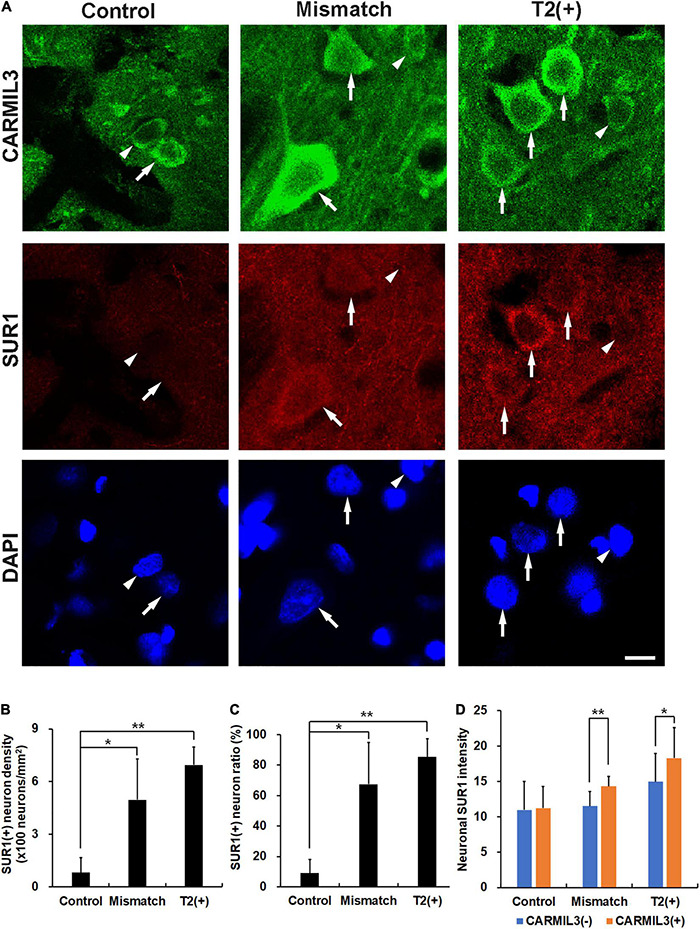
Expression of sulfonylurea receptor 1 (SUR1) in CARMIL3(+) neurons. **(A)** CARMIL3(+) neurons (arrows) had higher SUR1 intensity than CARMIL3(−) ones (arrowheads) in the mismatch and T2(+) regions after pMCAO. On the contrary, there was a low expression of SUR1 in the neurons at the control regions. **(B,C)** SUR1(+) neuron densities were significantly different among the three regions (*p* = 0.009), and so were SUR1(+) neuron ratios [the number of SUR1(+) neurons divided by that of all neurons] (*p* = 0.015). Higher SUR1(+) neuron densities **(B)** and neuron ratios **(C)** were noted in the mismatch and T2(+) regions compared with the control areas (all *p* < 0.05). **(D)** CARMIL3(+) neurons had significantly higher SUR1 intensity than CARMIL3(−) neurons in the mismatch and T2(+) regions. *p < 0.05; **p < 0.01. *N* = 4 rats in each group. Scale bar = 10 μm.

As CARMIL3 immunoreactivity was associated with larger neuronal cell bodies and neurites, we hypothesized that cell edema induced by ischemia underlay this phenomenon. We investigated the expression of SUR1, a regulator of a non-selective cation channel that is opened by depletion of ATP and contributes to cell edema (Simard et al., 2006), and found its expression in the neurons to be higher in the T2(+) and mismatch regions than those in the control region (all *p* < 0.05) (Figures 6A–C). In the mismatch and T2(+) regions, CARMIL3(+) neurons had higher SUR1 intensity than CARMIL3(−) neurons (*p* < 0.01 and *p* < 0.05, respectively) (Figure 6D). Overall, neuronal SUR1 intensity was significantly affected by regions (*p* = 0.003) and CARMIL3 positivity (*p* = 0.0003) in two-way ANOVA analysis.

### Association of Capping Protein Regulator and Myosin 1 Linker 3 With Impaired Autophagy

**FIGURE 7:**
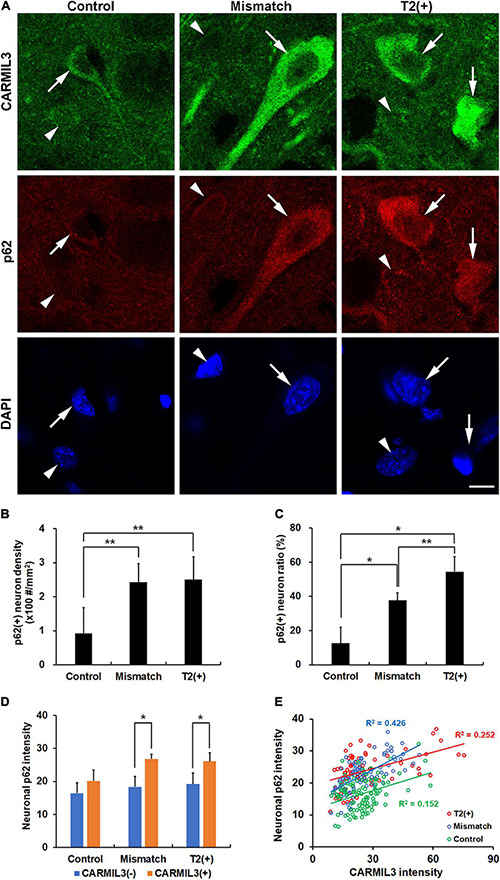
Association of CARMIL3 with impaired autophagy. **(A)** Immunostaining results of CARMIL3 and p62, an autophagy marker, in the T2(+), mismatch, and control areas. CARMIL3(+) neurons (arrows) were characterized by high expression of p62 compared with CARMIL3(−) neurons (arrowheads) in the T2(+) and mismatch regions. **(B,C)** p62(+) neuron densities **(B)** and ratios **(C)** in the three regions. The p62(+) neuron densities were significantly different among the three regions (p = 0.004), with a similar pattern in p62(+) neuron ratios [the number of p62(+) neurons divided by total number of neurons] (p = 0.018). The p62(+) neuronal densities and ratios in the mismatch and T2(+) regions were higher than those in the control areas (all *p* < 0.05). **(D)** Neuronal p62 intensity in CARMIL3(+) and CARMIL3(−) neurons in the three regions. CARMIL3(+) neurons had significantly higher p62 intensity than CARMIL3(−) neurons in the T2(+) and mismatch regions (both *p* < 0.05). **(E)** Correlation of p62 intensity with CARMIL3 intensity of all neurons in one image of each region in four rats. There was a significant association between p62 and CARMIL3 intensities in each region (all *p* < 0.001). In addition, there were significant differences in the regression slopes between the T2(+) and mismatch regions (*p* = 0.004) and between the mismatch and control regions (*p* = 0.017). *p < 0.05; **p < 0.01. Scale bar = 10 μm.

Given that (1) autophagy plays an important role in clearing toxic cellular wastes or damaged organelles recognized and bound by p62 (Jeong et al., 2020), and (2) p62 accumulation is considered a sign of impaired autophagy, we investigated the relationship between CARMIL3 and p62 in these brain areas. There was a parallel expression of p62 with CARMIL3, that is, a higher p62 expression was colocalized with a higher CARMIL3 expression in the neurons at the T2(+) and mismatch regions (Figure 7A). The p62(+) neuron densities were significantly different among the three regions (*p* = 0.004), which were similarly observed in p62(+) neuron ratios [the number of p62(+) neurons divided by total number of neurons] (*p* = 0.018). The p62(+) neuron densities and ratios were higher in the T2(+) and mismatch regions than in the control areas (all *p* < 0.05) (Figures 7B,C). CARMIL3(+) neurons had significantly higher p62 intensities than CARMIL3(−) neurons in the T2(+) and mismatch regions (both *p* < 0.05) (Figure 7D). Moreover, the correlations between p62 and CARMIL3 intensity in individual neurons were significant in the T2(+), mismatch, and control regions, respectively (all *p* < 0.001); these relationships were graded, that is, a significant difference was present in the regression slopes between the T2(+) and mismatch regions (*p* = 0.004) and between the mismatch and control regions (*p* = 0.017) (Figure 7E).

### Association of Capping Protein Regulator and Myosin 1 Linker 3 With Oxidative Stress

**FIGURE 8:**
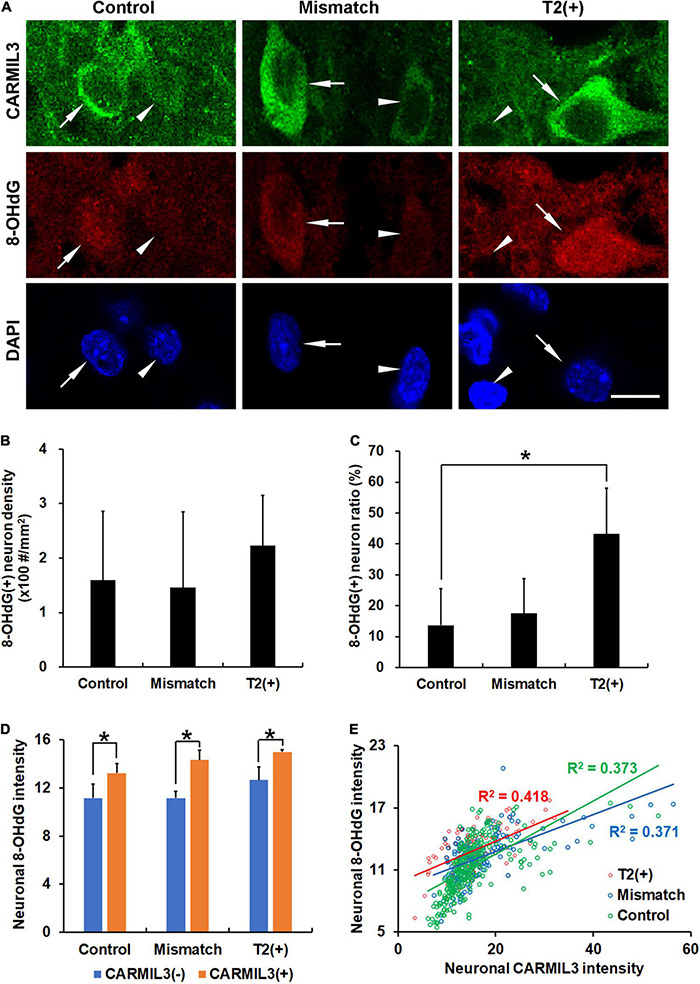
Association of CARMIL3 with oxidative stress. **(A)** Representative images of double-labeling for CARMIL3 and 8-OHdG in the T2(+), mismatch, and control regions. CARMIL3(+) neurons (arrows) tended to have higher 8-OHdG intensity than CARMIL3(−) neurons (arrowheads). **(B,C)** 8-OHdG(+) neuron density **(B)** and 8-OHdG(+) neuron ratio [the number of 8-OHdG(+) neurons divided by total number of neurons] **(C)** in these three regions. The 8-OHdG(+) neuron ratios were significantly different among the three regions (*p* = 0.027). 8-OHdG(+) neuron ratios, but not densities, were higher in the T2(+) areas than in the contralateral areas (*p < 0.05). **(D)** Quantitative comparison of neuronal 8-OHdG intensity between CARMIL3(+) and CARMIL3(−) neurons. CARMIL3(+) neurons had higher 8-OHdG intensities than CARMIL3(−) neurons in all three regions (all *p* < 0.05). **(E)** Correlation between 8-OHdG and CARMIL3 intensities of all neurons in one image of each region in four rats. There were significant associations between neuronal 8-OHdG and CARMIL3 intensities in all three regions (all *p* < 0.001). There was a significant difference in the regression slopes between the mismatch and control regions (*p* = 0.003). Scale bar = 10 μm.

To explore the significance of the findings above, we tested the hypothesis that the changes in CARMIL3 and impaired autophagy in the T2(+) and mismatch regions were secondary to oxidative stress. 8-OHdG is a product of DNA peroxidation, indicating cellular oxidative stress (Nagayama et al., 2000). On double-labeling staining of CARMIL3 and 8-OHdG, high CARMIL3 expression was colocalized with high 8-OHdG expression (Figure 8A). The ratios of 8-OHdG(+) neurons [the number of 8-OHdG(+) neurons divided by total number of neurons] were significantly different among the three regions (*p* = 0.027), and were higher in the T2(+) area than in the control region (*p* = 0.047) (Figures 8B,C). CARMIL3(+) neurons had higher 8-OHdG intensities than CARMIL3(−) neurons in the T2(+), mismatch, and control regions (all *p* < 0.05) (Figure 8D). Furthermore, neuronal CARMIL3 intensities were significantly associated with 8-OHdG intensities in all three regions (*p* < 0.001). The regression slopes between the mismatch and control regions were significantly different (*p* = 0.003), while the slopes were similar between the T2(+) and mismatch regions (Figure 8E).

### Capping Protein Regulator and Myosin 1 Linker 3 Expression in Human and Rat Infarct Tissues at Subacute Stage

**FIGURE 9:**
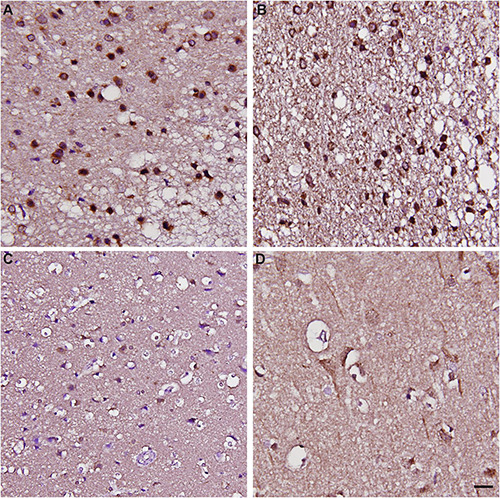
Immunohistochemical staining of CARMIL3 in human infarct brains and stroke-free autopsied brains. **(A–C)** CARMIL3 expression in human infarct brain tissues. In the region most sever ely infarcted (right lower part of **A**), there were fewer neurons, with all the residual neurons strongly positive for CARMIL3. In the area surrounding the sever ely infarcted region, most neurons had strong CARMIL3 staining in their nuclei and cytoplasm. In the region with the least infarction severity (left upper part of **A**), most neurons had normal nuclear morphology with faint CARMIL3 staining mainly in the cytoplasm. **(D)** Relatively low CARMIL3 expression in the neurons of stroke-free autopsied brains. Scale bar = 20 μm.

To validate whether such a pattern of CARMIL3 expression is an ischemic signature of neurons in the human brain, we examined the resected infarcted human brain tissues and stroke-free autopsied brain tissues with CARMIL3 immunohistochemical staining (Figure 9). Clinical information of the three stroke patients is shown in Table 2, and the surgeries were performed between 2 and 4 days after stroke. The most severely infarcted areas (the right lower quadrant of Figure 9A and the right half of Figure 9B) had fewer neurons, but all the residual neurons were strongly positive for CARMIL3. In the areas surrounding the severely infarcted region, most neurons had strongly CARMIL3-stained nuclei and cytoplasm. In the region with the least infarction severity (the left upper quadrant of Figures 9A,C), most neurons had normal nuclear morphology with faint CARMIL3 staining, mainly in the cytoplasm. In contrast, the brain sections of control subjects had lower CARMIL3 intensity in the cortical neurons (Figure 9D).

**TABLE 2:** Clinical information of the human stroke cases.

	Case A	Case B	Case C
Age (years)	40	62	66
Sex	Male	Female	Female
Infarct region	Left MCA territory	Right ICA territory	Left MCA territory
Occluded artery	Left distal ICA to MCA	Right ICA	Left MCA
Craniectomy and	4 days	2 days	3 days
strokectomy timing			
since stroke onset			
Strokectomy areas	Frontal, temporal	Frontal, temporal	Frontal, temporal
Stroke subtype	Cardioembolism	Cardioembolism	Cardioembolism

*MCA, middle cerebral artery; ICA, internal carotid artery. The case numbers were corresponding to the subfigure numbers of Figure 9.*

**FIGURE 10:**
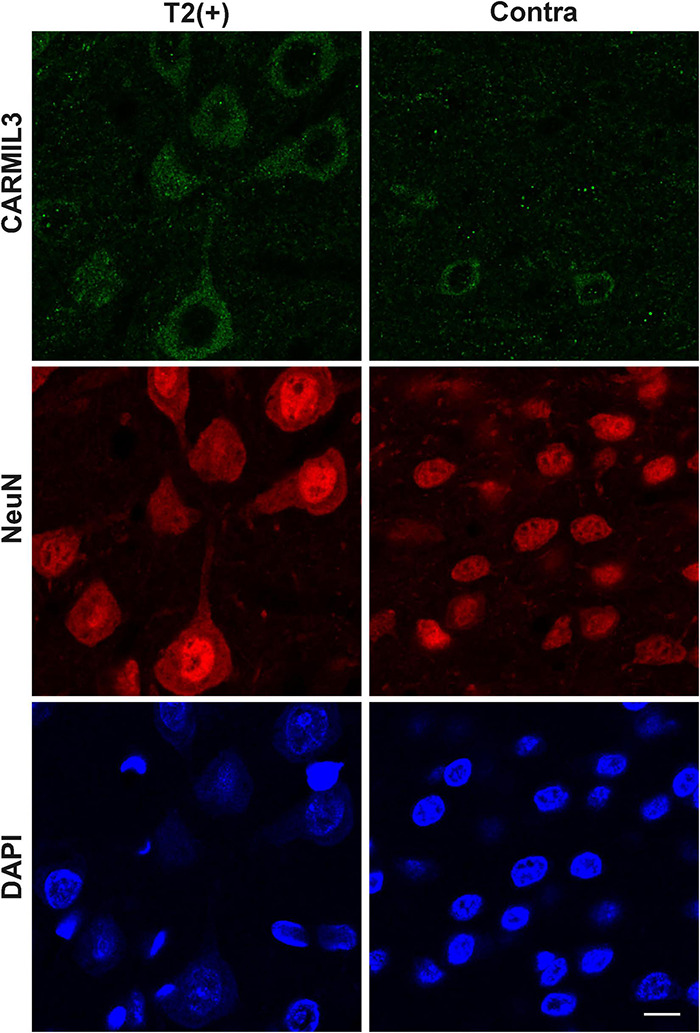
Immunofluorescence staining of CARMIL3 in rat brains after pMCAO for 3 days. CARMIL3 expression at T2(+) and contralateral regions in the rat brains after pMCAO for 3 days. There were only minimal mismatch areas, thus they were not assessed. Many neurons in the T2(+) regions were swelling or deformed. CARMIL3 was expressed in more neurons at T2(+) regions compared with the contralateral regions, although the signal intensities of CARMIL3 in all neurons at this stage were very low compared with those in the T2(+) regions within 4.5 h after pMCAO [comparison of CARMIL3(+) count: *p* = 0.011]. Scale bar = 10 μm.

To certify the findings of CARMIL3 expression in human infarcted brain tissues at subacute stage, the rat brains of pMCAO for 3 days were assessed by CARMIL3 immunofluorescence staining. The brain MRI at 24 h showed only minimal mismatch between DWI(+) and T2(+) regions (*p* > 0.05). Many neurons in the T2(+) regions were swelling or deformed (Figure 10). CARMIL3 was expressed in more neurons at T2(+) regions compared with the contralateral regions, although the signal intensities of CARMIL3 in all neurons at this stage were very low [coded as CARMIL3(−) based on the criteria in Figure 3] compared with those in the T2(+) regions within 4.5 h after pMCAO (*p* = 0.011).

### Capping Protein Regulator and Myosin 1 Linker 3 Expression in Primary Rat Cortical Neuron Culture

**FIGURE 11:**
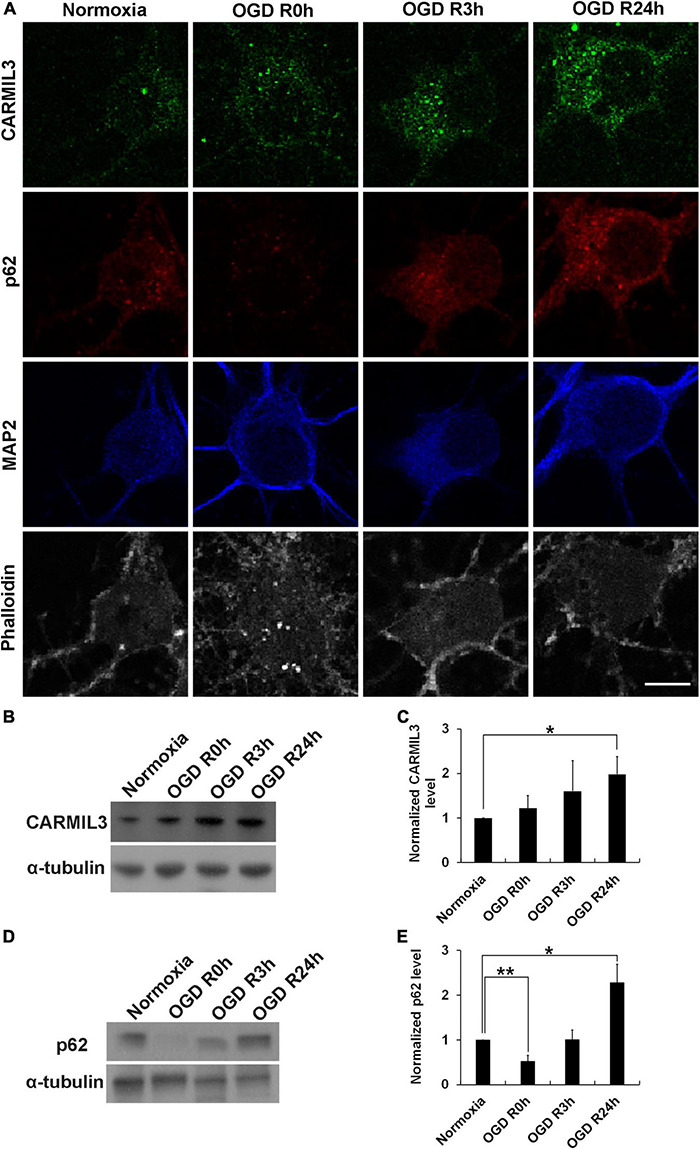
CARMIL3 expression in rat primary cortical neuronal culture. **(A)** Immunofluorescence staining of CARMIL3, p62, MAP2, and phalloidin in primary rat cortical neuron culture after oxygen-glucose deprivation (OGD). CARMIL3 was obviously expressed as a beaded pattern in the neurons at OGD R3h and OGD R24h; p62 had increased expression in neurons at the two points in time. There was more phalloidin staining at OGD R3h, indicating more expression of filamentous actin. **(B,C)** Western blot for CARMIL3 in neuronal culture showed that OGD R24h had significantly increased CARMIL3 levels than the normoxia group (normalized expression level 1.98 vs. 1, *p* = 0.025, *n* = 3; *p* = 0.065 in comparison among the 4 groups). **(D,E)** Western blot for p62 in neuronal culture revealed lower expression in the OGD R0h group while higher expression in the OGD R24h group compared with that in the normoxia group. *p < 0.05; **p < 0.01. Scale bar = 10 μm.

Given the exclusive neuronal expression of CARMIL3, we investigated whether an *in vitro* model of neuronal ischemia could replicate the expression of CARMIL3 by conducting standard OGD experiments on primary rat cortical neuron cultures. Immunofluorescence staining showed that CARMIL3 was expressed as a beaded pattern in the neurons at reperfusion 3 h (OGD R3h) and 24 h (OGD R24h) after OGD (Figure 11A), with concomitantly increased expression of p62 in the neurons. The staining intensity of phalloidin was increased at OGD R3h, indicating an upregulated expression of filamentous actin. Western blotting revealed higher expression of CARMIL3 in the OGD R24h group than that in the normoxia group (normalized expression level 1.98 vs. 1, *p* = 0.025, *n* = 3) (Figures 11B,C). The expression level of p62 was lower in the OGD R0h group (normalized expression level 0.52 vs. 1, *p* = 0.007) while higher in the OGD R24h group (normalized expression level 2.28 vs. 1, *p* = 0.012) compared with that in the normoxia group (Figures 11D,E), possibly suggesting activated autophagy after ischemia followed by impaired autophagy after reperfusion for 24 h.

## Discussion

This report documented CARMIL3 as a molecular signature of ischemic neurons in the mismatched brain areas between DWI and T2 imaging patterns. The upregulation of CARMIL3 in neurons reflects cell edema, impaired autophagy, and oxidative stress. Although DWI-T2 and DWI-FLAIR mismatch on MRI after ischemic stroke serves as an indicator of ischemia within 4.5 h, the molecular patterns and cellular stress in this region have not been systemically elucidated. In this study, the mismatch regions referred to the area with a difference in imaging patterns between positive DWI and T2 in rats. We focused on the mismatch and T2(+) regions after focal cerebral ischemia for less than 4.5 h using pMCAO and tMCAO models to mimic clinical ischemic stroke without and with recanalization therapy, respectively. There was no obvious cellular loss in the T2(+) and mismatch regions in the early stage of ischemia; however, apoptosis was observed in these areas, with necroptosis only in the T2(+) area. Proteomics experiments identified the protein with the highest expression in the T2(+) region to be CARMIL3. In the mismatch and T2(+) regions, CARMIL3(+) neurons increased in number and had (1) swollen cell bodies and neurites with increased SUR1 expression, (2) accumulation of p62, indicating impaired autophagy, and (3) increased 8-OHdG, indicating oxidative stress. The increased expression of CARMIL3 after ischemia in rat brains was consistent with that in infarcted human brain tissues and primary rat cortical neuron cultures. These results provide a molecular signature of ischemic neurons in the T2(+) and mismatch regions within a few hours of ischemia.

### The Cellular Pathology in the Mismatch and T2(+) Regions

Given the lack of information on the pathological and molecular expression patterns in the mismatch regions after ischemic stroke, we first demonstrated that the cell and NeuN(+) neuronal densities did not change significantly in the T2(+) and mismatch areas. NeuN is a specific neuronal marker expressed in most types of neurons in the central nervous system (Gusel’nikova and Korzhevskiy, 2015). Some neurons, especially the damaged ones, might lose NeuN immunoreactivity due to decreased synthesis or changed immunoreactivity of NeuN, which may underestimate neuron density (Unal-Cevik et al., 2004). We showed that some neurons had a faint NeuN staining in the T2(+) region, indicating the transition to the absence of NeuN immunoreactivity. In addition to brain ischemia, various brain pathologies such as irradiation injury, poison toxicity, and viral infection also reduce NeuN immunoreactivity in neurons (Collombet et al., 2006; Wu et al., 2010; Hahn et al., 2015). Altogether, this study provides evidence of changes in the pathology of ischemic neurons with a short ischemic duration.

### Capping Protein Regulator and Myosin 1 Linker 3 Expression in the Mismatch and T2(+) Areas

Based on the proteomic analysis, we identified CARMIL3 as a molecular candidate of ischemic neurons in the mismatch and T2(+) areas, with increased expression in the neuronal cytoplasm and neurites. CARMIL3 expression was increased in both the mismatch and T2(+) areas, although the ratios varied in different measurements: biochemical proteomics vs. immunofluorescence staining. CARMIL3(+) neurons were most abundant in the mismatch region in the pMCAO group, with a similar but less prominent pattern in the tMCAO-90 group, which further decreased in the tMCAO-30 group, indicating that reducing ischemic burden by early recanalization significantly reduced the CARMIL3 changes in ischemic neurons. Moreover, the larger sizes of neuron cell bodies and neurites in the T2(+) and mismatch regions were consistent with the cytotoxic edema revealed by positive DWI signal, which was also associated with CARMIL3 expression. Furthermore, the increased SUR1 expression in the T2(+) and mismatch regions, more obvious in the T2(+) region, suggests that the T2(+) region had higher edema severity than the mismatch area and was strongly associated with CARMIL3 expression. Furthermore, the mismatch area had more CARMIL3(+) neurites than the T2(+) area, probably because the ischemic damage in the T2(+) area affected neurite integrity (Ito et al., 2006). The low expression of CARMIL3 in the T2(+) regions in 3 days after pMCAO showed that the increase of CARMIL3 within 4.5 h of ischemia was a transient phenomenon.

Capping protein regulator and myosin 1 linker 3 is one of three members of the CARMIL family that are highly conserved in vertebrates (Stark et al., 2017). CARMILs inhibit the actin-binding activity of capping protein with high affinity, and each CARMIL isoform has its specific function: CARMIL1 regulates actin dynamics in lamellipodia to control protrusion, ruffling, and vesicle-mediated endocytosis; CARMIL2 is important for cell polarity (Liang et al., 2009); and CARMIL3 has multiple protein-protein interactions in immunoprecipitation experiments indicating potentially diverse functions (Wang et al., 2020), although this topic has never been extensively explored. The known functions of CARMIL3 include enhancing tumor metastasis through promoting cell migration, adhesion, invasion, and metastatic colonization (Wang et al., 2020); promoting cell migration in immune cells (Liang et al., 2009); and regulating synapse maturation in developing perinatal brains (Spence et al., 2019). This study shows, for the first time, that CARMIL3 serves as a molecular signature of ischemic neurons. The neuronal swelling may be related to the volume-regulatory function of actin. CARMILs prohibit capping protein from binding to actin filament and rapidly uncap the capping protein-capped filament, thereby promoting the elongation of actin filament (Yang et al., 2005). Moreover, actin synthesis and polymerization can develop rapidly to restrain cell swelling and control several ion channels in the cell edema induced by hypo-osmotic environment in astrocytes (Morán et al., 1996). The findings of this study indicate that CARMIL3(+) neurons might be under oxidative stress with impaired autophagy after ischemic damage, which might endanger their survival. Further studies are warranted to confirm the role of CARMIL3 in neurons undergoing ischemic injury. Given that CARMIL3 is increased in the infarct brain regions starting from the early hours, it may be detectable in the blood of stroke patients, which possibly has the potential as a biomarker of stroke severity. With the elucidation of CARMIL3 functions, the modulation of CARMIL3 may serve as a new strategy for acute stroke to mitigate the ischemic neuronal injury especially for those without recanalization therapy.

This study had some limitations. First, there was a latency of 30–55 min between MRI completion and perfusion fixation, which may allow ischemic lesion progression to occur in this period. However, another set of animals (*n* = 4) that received pMCAO followed by repeated MRI acquisition at an interval of 40 min showed no interval change in the volumes of mismatch and T2(+) areas (*p* = 0.368 and 0.641, respectively). Second, neurons in different cortical regions may have different phenotypes, which may interfere with the comparison between different areas. To circumvent this issue, we reduced its potential influence by using the contralateral region in the corresponding area as a control. Third, there was a lack of experimental data on the functions of CARMIL3 in cerebral ischemia. Interventional approaches will be needed to address this issue. Fourth, the staining of CARMIL3 in human tissues did not match the time frame of the animal models. The mass effect of large infarction leading to craniectomy usually occurs during 2 to 4 days after stroke. This time course makes it impossible to obtain the human infarcted brain tissues within early hours after onset. Nevertheless, our data of human brain tissues showed that the expression of CARMIL3 was also increased in human ischemic brain and corresponding to the severity of infarction. Fifth, naïve animals instead of sham group were used as controls in the proteomics experiments, rising the concern that the changes of CARMIL3 might be induced by surgical effect other than vessel occlusion. However, the significantly lower CARMIL3(+) neuron densities in the sham group compared with the three regions of pMCAO group suggesting the CARMIL3 changes after pMCAO mostly attributed to vessel occlusion. Therefore, in the proteomics and immunofluorescence experiments, the contralateral corresponding regions served as controls which had the same surgical exposure with the ischemic regions except for vessel occlusion.

## Conclusion

In conclusion, this study identified CARMIL3 as a molecular signature of ischemic neurons and a pathological substrate in the mismatch and T2(+) regions within 4.5 h of MCAO. The neurons in the mismatch and T2(+) areas underwent apoptosis, while necroptosis was observed in the T2(+) areas. CARMIL3 was highly expressed in the neurons in these two ischemic regions, particularly the mismatch region, with significant cellular edema in neuronal cell bodies and neurites, impaired autophagy, and increased oxidative stress. These findings may offer a new direction for therapy during cellular stress in early hours after cerebral ischemia.

## Data Availability Statement

The datasets presented in this study can be found in online repositories. The names of the repository/repositories and accession number(s) can be found below: the proteomics data have been uploaded onto ProteomeXchange via the PRIDE database. Project accession: PXD027890.

## Ethics Statement

The studies involving human participants were reviewed and approved by Research Ethics Committee of the National Taiwan University Hospital (reference number: 201112173RIC for infarcted human brain tissue; 201908065RINA for normal cadaver brain tissue). The patients/participants provided their written informed consent to participate in this study. The animal study was reviewed and approved by Institutional Animal Care and Use Committee of the National Taiwan University College of Medicine (reference number: 20200126).

## Author Contributions

S-JY performed the animal surgeries and the staining experiments of animal and human specimens, analyzed and interpreted the data, and was a major contributor in writing the manuscript. P-HH performed the proteomics experiments. T-YY assisted the MRI examinations and the western blotting. W-KY assisted in the analysis of microscopic images. K-PC offered the human cadaver brain tissue sections. C-SC performed the cell culture experiments. S-CT, L-KT, and J-SJ offered suggestions for revising the manuscript. S-TH contributed to study conception and design, revision the manuscript, supervision and funding acquisition. All authors contributed to the article and approved the submitted version.

## Conflict of Interest

The authors declare that the research was conducted in the absence of any commercial or financial relationships that could be construed as a potential conflict of interest.

## Publisher’s Note

All claims expressed in this article are solely those of the authors and do not necessarily represent those of their affiliated organizations, or those of the publisher, the editors and the reviewers. Any product that may be evaluated in this article, or claim that may be made by its manufacturer, is not guaranteed or endorsed by the publisher.

## Abbreviations

CARMIL3: capping protein regulator and myosin 1 linker 3
DWI: diffusion weighted image
FLAIR: fluid-attenuated inversion recovery
GFAP: glial fibrillary acidic protein
MAP2: microtubule associated protein 2
MCAO: middle cerebral artery occlusion
OGD: oxygen-glucose deprivation
pMCAO: permanent middle cerebral artery occlusion
pRIP3: phosphorous receptor-interacting protein kinase 3
tMCAO: transient middle cerebral artery occlusion
SUR1: sulfonylurea receptor 1
8-OHdG: 8-hydroxy-2 ′ –deoxyguanosine.
